# Motivational Interviewing for Loved Ones in Early Psychosis: Development and Pilot Feasibility Trial of a Brief Psychoeducational Intervention for Caregivers

**DOI:** 10.3389/fpsyt.2021.659568

**Published:** 2021-04-01

**Authors:** Emily R. Kline, Heather Thibeau, Aliyah S. Sanders, Kelly English, Beshaun J. Davis, Alicia R. Fenley, Matcheri S. Keshavan

**Affiliations:** ^1^Department of Psychiatry, Beth Israel Deaconess Medical Center, Boston, MA, United States; ^2^Department of Psychiatry, Harvard Medical School, Boston, MA, United States; ^3^Boston Medical Center, Boston, MA, United States; ^4^Department of Psychiatry, Boston University School of Medicine, Boston, MA, United States; ^5^Department of Psychology, Georgia State University, Atlanta, GA, United States; ^6^Massachusetts Department of Mental Health, Boston, MA, United States; ^7^Department of Psychology, Boston University, Boston, MA, United States

**Keywords:** schizophrenia, first episode psychosis, motivational interviewing, family, caregiver, clinical trial, feasibility studies

## Abstract

Treatment delay and non-adherence in first episode psychosis is a pressing public health problem. Ambivalence regarding psychiatric intervention and labeling among young people with psychosis is a contributing factor. For these individuals, caregivers often facilitate the pathway to care and support ongoing engagement and adherence. Caregivers describe distress and burden associated with this role. This manuscript describes the development and pilot feasibility testing of a motivational interviewing-derived communication training for caregivers of individuals with untreated or under-treated early course psychosis. Individuals with lived experience were consulted in the intervention development process. The training consisted of four 60-min sessions teaching the philosophy and basic skills of motivational interviewing as well as two brief practice calls. Feasibility was assessed with regard to study enrollment, retention, and completion. Satisfaction was assessed through the Client Satisfaction Questionnaire and qualitative feedback. Thirty-one caregivers consented to this pilot feasibility trial and participated via telehealth over the course of 5 months. Intervention completion and reported satisfaction were high, with 94% of consented participants completing at least three training sessions and 84% reporting that they would “definitely” recommend the training to a friend in similar circumstances. There were no between-clinician differences in MILO session attendance (*F*_[2]_ = 0.53, *p* = 0.596) or satisfaction total scores (*F*_[2]_ = 1.03, *p* = 0.371). Brief motivational interviewing skills training appears to be a feasible and valued intervention for caregivers of individuals with poorly managed early course psychosis.

**Clinical Trial Registration:** ClinicalTrials.gov Identifier: NCT04010747

## Introduction

First episode psychosis (FEP) often represents a time of crisis for young people and their families. Although some psychoses are self-limiting, more often these symptoms portend a potentially chronic and disabling psychiatric disorder such as schizophrenia. Meta-analyses indicate that coordinated multidisciplinary intervention early in the course of psychosis, including family intervention, can alleviate symptoms and restore functioning more effectively than “standard” community treatment (1, 2). The Recovery After Initial Schizophrenia Episode research initiative established that coordinated specialty care for FEP could be feasibly implemented in the United States, and is more effective than treatment as usual for decreasing clinical symptoms, improving quality of life, and increasing participation in school and work (3). However, this study found that many patients entered care with long duration of untreated psychosis (DUP), adding to a consensus that treatment benefits are generally far greater for psychosis patients with shorter as opposed to longer DUP (3–5).

Treatment delay and non-adherence in FEP is a pressing public health problem. A review of privately insured adolescents and young adults in the US showed that 62% of young people in the US with FEP filled no outpatient prescriptions, and 41% received no outpatient psychotherapy, in the year following their index diagnosis (6). Among those who do encounter specialized FEP outpatient care, high attrition is a common problem, with 20-50% of individuals initially enrolled in first episode programs dropping out (7). The reasons underlying long DUP and poor engagement in care are myriad. Many individuals experiencing psychosis are reluctant to seek or adhere to mental health treatments due to lack of insight and/or concerns about the usefulness of psychiatric interventions (8, 9). Young adults may be torn between distress and dissatisfaction relating to their symptoms and functioning on the one hand, and mistrust of mental health providers, treatments, and labels on the other (10). Family members and other loved ones often endure confusion and distress as they endeavor to convince the individual with psychosis (IP) to accept and utilize psychiatric services (11–13).

Motivational interviewing (MI) is a well-established strategy for facilitating behavior change across a wide range of treatment targets, including enhanced adherence to treatment. The theme of MI is non-judgmental exploration of ambivalence regarding behavior change (14). MI is not didactic or confrontational; rather, it is a set of communication strategies designed to decrease defensiveness and rigidity. Clinician-delivered MI has been identified as effective for enhancing adherence once individuals with psychosis are involved in care (15, 16), and may also be useful for engaging those who are not yet interested in treatment (14). Several studies have found positive results in training and deploying non-professionals to use MI to influence target health behaviors such as substance use and diet (17, 18). Only one study to date has trained parents to use MI in the context of recent-onset schizophrenia; the authors reported that individuals whose caregivers learned MI used less cannabis and had less severe symptoms over the following 15 months than those whose families received routine care (19–21). MI-derived communication training for caregivers may represent a promising approach through which parents or other relatives may be able to improve relationships, decrease conflict, and influence a loved one's decision to seek care and adhere to treatment plans (20, 22–25).

The aim of the current study is to develop and test the feasibility of a brief MI-derived psychoeducational intervention for parents and other close contacts of individuals with early course psychosis who are sub-optimally engaged with treatment. The goal is not that the caregiver becomes a “therapist” to the individual with psychosis (IP), but rather that they learn and use MI-based communication strategies to decrease conflict in the relationship and play a more effective role in helping to connect the IP to relevant clinical services. The aim of this paper is to describe the development of the intervention and study procedures, determine the feasibility of the pilot protocol, and assess participants' satisfaction with the intervention.

## Materials and Methods

### Intervention Development

The author group conducted stakeholder interviews to inform the development of the “motivational interviewing for loved ones” (MILO) intervention. Consultants with lived experience were interviewed about the process of seeking care for themselves or their child, and their impressions of family needs during care initiations and transitions in general. We then attended formal trainings for providers offered by certified trainers in motivational interviewing (EK) and Community Reinforcement and Family Training (HT), a related evidence-based practice that teaches skills pertaining to behavior change and reflective listening to caregivers of individuals with substance use disorders. We also consulted with a Motivation Interviewing Network of Trainers-certified trainer (Angela Cooper) about the curriculum structure of MI training for clinical providers. After these meetings, we (EK, HT, AS, KE) reviewed our notes and impressions to reach a consensus on which core MI-consistent skills to include in the training. Once the core skills were identified (see Table 1), we created a manual for clinicians to use in MILO sessions. Clinicians were to both teach the MILO skills and also model them consistently during sessions by being fairly non-directive, for example asking open-ended questions and using reflections to help caregivers process their own ambivalence about using MILO skills or other dilemmas.

**Table 1:** Motivational interviewing for loved ones: session content.

Core skills	• The “spirit” of motivational interviewing • Learning not to fix or minimize others' problems • Reflections • Questions • Affirmations • Raising difficult topics • Obtaining permission before giving advice
Session structure	• Session 1: Review the individual with psychosis's current treatment status, well-being, treatment history, and relationship with the participant. If needed, offer information about relevant treatment (e.g., coordinated specialty care). Inquire about impact of illness on participant. Teach participant about the concept of motivational interviewing (MI) and the “spirit” of MI. • Session 2: Teach and practice reflections, open-ended questions, and affirmations • Session 3: Teach and practice raising difficult topics and asking permission before giving advice • Session 4: Review a conversation, plan a conversation, and/or devote more time to in-session practice

Concerns about feasibility and cultural relevancy were of foremost consideration in designing the content and duration of this intervention. In order to maximize feasibility and minimize burden to participants, we prioritized keeping both the intervention and the assessment battery brief. The intervention was designed to be completed in four 45–60 min sessions, and the assessment battery in 25 min or less. Additionally, we strove to create a culturally conscious intervention informed by diverse needs and perspectives that would not need to be “adapted” at a later point to fit the cultures and concerns of non-white families (26). To do so, we consulted stakeholders representing a diverse range of cultural backgrounds and relevant lived experiences throughout intervention development, minimized the use of psychological jargon in the manual, and chose images for the manual that represented diverse families.

Just as recruitment for the study was beginning (February 2020), the COVID-19 pandemic struck the United States and non-essential in-person activities were suspended indefinitely at the study site (Beth Israel Deaconess Medical Center). At this point, MILO was re-designed as a telehealth intervention, and the manual was translated into a digital slide deck that would be shared over the screen with caregivers during a video-conference meeting.

MILO facilitators included the first author (EK) as well as two additional clinicians (BD, AF). The first author and senior clinician (EK) is a licensed doctoral level psychologist with training in both psychosis treatment and motivational interviewing. The other study clinicians were a post-doctoral psychology fellow (BD) and an advanced student in a clinical psychology doctoral program (AF). Clinicians trained in the intervention by reviewing the manual with the first author, observing her in three MILO sessions, and discussing cases with her weekly. Fidelity was assessed by each clinician documenting which MILO-relevant skills and themes were covered in each session and reviewing these in supervision sessions.

### Procedures

Eligibility requirements for caregiver-participants were: age 18 or older, able to communicate in English, a primary caregiver and/or close contact who has ≥20 h weekly contact with an IP, and able to provide informed consent. Additionally, in order for caregivers to be eligible, the IP had to be 15–35 years old, diagnosed with a DSM-5 affective or non-affective psychotic disorder by a health professional OR have observable symptoms or behaviors (e.g., responding to internal stimuli, describing delusional ideas, or showing grossly disorganized speech or behavior) indicating psychosis, with onset of observed symptoms or first psychosis diagnosis within past 5 years. IP were either untreated or not optimally engaged in outpatient treatment (e.g., not adhering to prescribed medications, using substances in conflict with the treatment plan, or refusing to meet with providers). Due to the inevitable diagnostic uncertainty of relying only on caregiver report, study staff attempted to obtain collateral diagnostic information from another source when the diagnosis seemed very unclear. After each participant completed MILO sessions, the study clinician revisited the most likely diagnoses to confirm the presence of recent-onset psychosis.

The recruitment goal for the feasibility phase of the study was set at thirty. To recruit participants, the study's first author sent study information to clinicians and referral coordinators at FEP programs in the Boston area as well as through a national (U.S.) early psychosis-focused listserv. Clinicians and referral coordinators were encouraged to let potential participants know about the study depending on their clinical judgment and institutional policies.

This protocol was reviewed and approved by the Beth Israel Deaconess Medical Center institutional review board. Potential participants were screened for eligibility over the phone. If they were eligible, they then provided verbal informed consent via telephone. Self-report assessments were emailed to participants via a secure Redcap survey link. Participants were then asked to schedule a brief call with research staff for a pre-intervention recorded “real-play” in which research staff described a personal dilemma and asked participants to discuss it with them for 10 min. Once all pre-intervention assessments were completed, participants scheduled an initial session with a study clinician. All MILO sessions were conducted using a secure telehealth platform called Starleaf (as required by the institutional review board). After the intervention was concluded, participants recorded a second “real-play” and were emailed surveys at 0-, 8-, and 12-weeks following intervention completion. Participants were reimbursed for completing assessments ($25 per time point). No reimbursement was provided for attending MILO sessions.

### Measures

The following domains were selected to measure MILO feasibility: number/pace of inquiries, percent of inquiries eligible for participation, percent of eligible trial candidates who enrolled (the goal was two participants per month), intervention completion (number of sessions attended), assessment completion, and participant satisfaction. Participants were assessed in their first MILO session by a clinician to determine whether they met criteria for an adjustment disorder as a consequence of their loved one's psychotic illness, using the adjustment disorder section of the Structured Clinical Interview for DSM-5 (27). Participant satisfaction was measured at the post-intervention assessment via a seven-item version of the Client Satisfaction Questionnaire (28) (one original item about returning for additional services was omitted). We also surveyed participants on whether they had tried using MILO skills with the IP. Participants were then prompted to respond to three open-ended questions: what they had found helpful about MILO, suggestions for improving MILO, and what barriers they encountered to MILO skills with the IP.

The following domains were selected to measure MILO effects and were administered at each of the four assessment time points: past-month treatment attendance and adherence by the IP (as reported by the caregiver); expressed emotion [measured via the Family Questionnaire; Wiedemann et al. (29)]; family conflict and cohesion [measured via the Conflict Behavior Questionnaire, Robin and Foster (30); and the Score-15, Stratton et al. (31), respectively]; self-efficacy [measures via the Parenting Self-Agency Measure, Dumka et al. (32); and the General Self-Efficacy Scale, Chen et al. (33)]; and stress [Perceived Stress Scale; Roberti et al. (34)]. To assess the extent to which participants were able to learn and demonstrate MI skills (i.e., target engagement), caregivers completed a 10-item test of their knowledge of MI concepts and an audio-recorded behavioral skill demonstration at the baseline and immediate post-intervention time points. The present study reports on the feasibility rather than the effects of MILO.

### Analyses

Feasibility targets and client satisfaction were assessed using descriptive analyses only. Differences in MILO completion rates and client satisfaction scores between study clinicians were assessed via one-way ANOVA using SPSS. Qualitative responses were reviewed by the first author, who conducted an inductive thematic analysis to summarize responses (35).

## Results

### Feasibility

**Figure 1:**
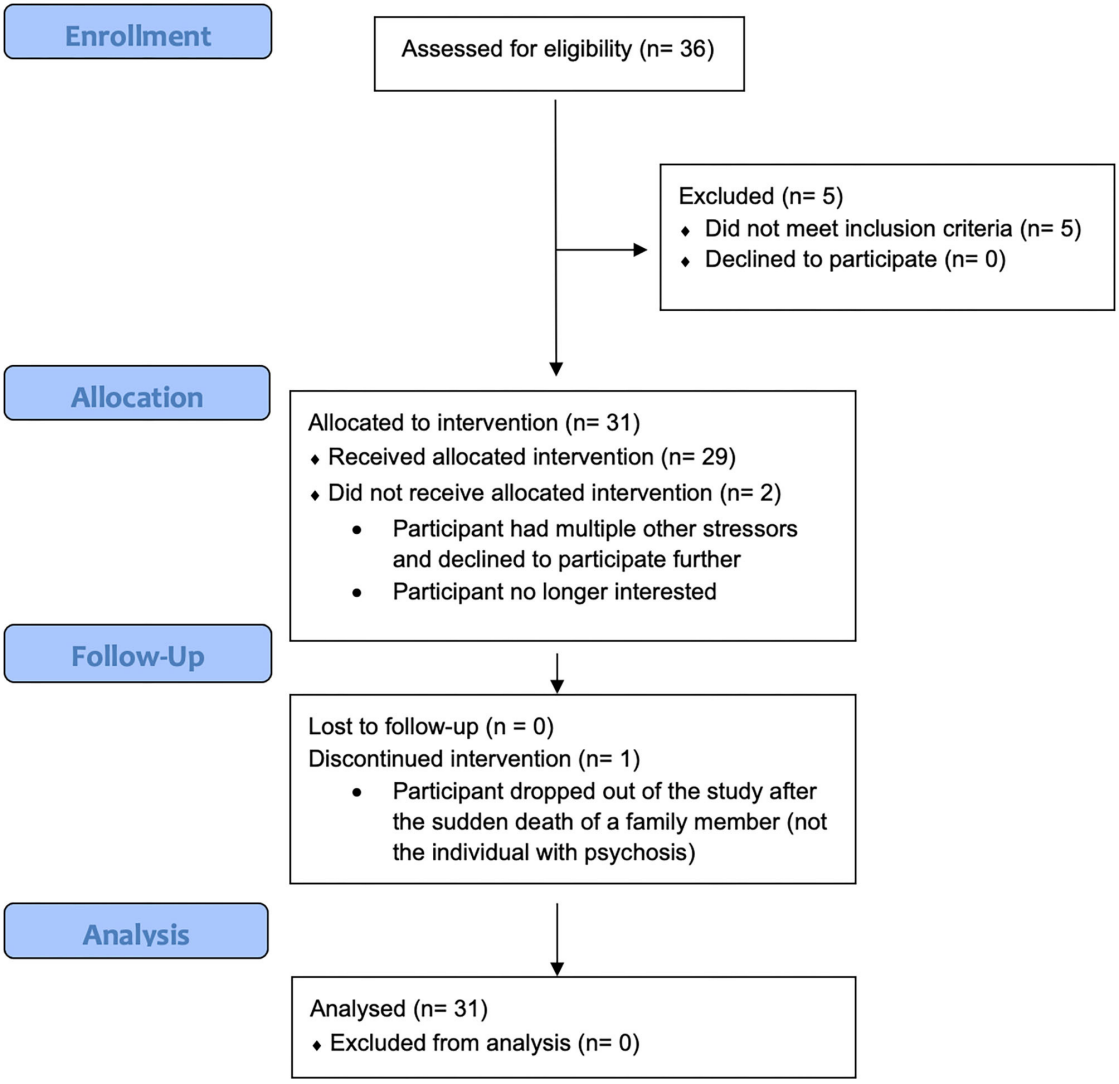
CONSORT flow diagram.

See Figure 1 for a CONSORT flow diagram reflecting this pilot feasibility trial.

A total of 43 people contacted study staff via email or phone to inquire about MILO participation between May 1, 2020 and September 11, 2020. Thirty-six were assessed by study staff and 31 were found to be eligible. Reasons for non-eligibility included not having a loved one with a psychotic disorder, duration of psychotic illness more than 5 years, IP older than 35, and being neither a primary caregiving nor spending at least 20 h weekly with the IP. Three participants who did not know their child's diagnosis were determined to meet eligibility criteria, since the participants each described a qualifying symptom that they and others had observed (delusional pre-occupation and/or disorganized speech) that had begun in the past five years, and the child had declined to participate in a psychiatric evaluation.

All eligible participants (*N* = 31) representing 25 families (some caregivers enrolled along with or subsequent to a co-parent) enrolled for the “phase 1” feasibility stage of this pilot trial. Participant characteristics are listed in Table 2. On average, the time from initial contact to informed consent was 3.6 days, and from consent to first MILO session was 16.7 days.

**Table 2:** Participant Characteristics (*N* = 31).

**Participant characteristics**	
Age	Range: 45-71 Mean (SD): 57.97 (7.43)
Gender	Male: 8 (26%) Female: 23 (74%)
Relationship to individual with psychosis	Parent: 31 (100%) Other: 0 (0%)
Residing with individual with psychosis	Yes: 19 (61%) No: 12 (39%)
Race	White: 25 (81%) Black: 1 (3%) Asian: 3 (10%) Other: 1 (3%) Prefer not to say: 1 (3%)
Ethnicity	Hispanic/Latino: 1 (3%) Not Hispanic/Latino: 30 (97%)
Immigration history	Born in United States: 26 (84%) Born elsewhere: 5 (16%)
Educational attainment	High school diploma or higher: 31 (100%) Bachelor's Degree or higher: 25 (81%)
Adjustment disorder diagnosis	Adjustment disorder: 14 (45%) No adjustment disorder: 14 (45%)^a^ Missing: 3 (10%)
**Characteristics of Individuals with Psychosis (as reported by participant)**	
Age	Range: 16-30 Mean (SD): 23.13 (3.89)
Gender	Male: 26 (84%) Female: 4 (13%) Unknown: 1 (3%)
Diagnosis	Schizophrenia: 6 (19%) Schizoaffective disorder: 9 (29%) Schizophreniform disorder: 2 (6%) Bipolar disorder with psychotic features: 6 (19%) Clinical high-risk for psychosis: 1 (3%) Other unspecified psychosis: 4 (13%) Unknown: 3 (10%)
Co-occurring substance use	Yes, current: 21 (68%) Yes, past: 2 (6%) No: 6 (19%) Unknown: 2 (6%)
Duration of psychotic illness (years)^b^	Range: 0.25-4.67 Mean (SD): 2.10 (1.32)
History of psychiatric hospitalization	Yes: 23 (74%) No: 8 (26%)
Past-month psychiatric service utilization	Stayed overnight in hospital: 8 (26%) Visited emergency room: 10 (32%) Took any medication: 19 (61%) Took medication as prescribed: 10 (32%) Attended ≥1 outpatient appointment: 13 (42%)

a *Two participants who did not meet DSM-5 criteria for Adjustment Disorder disclosed that they had other established diagnoses of Major Depressive Disorder and Post-Traumatic Stress Disorder, respectively*.

b *N = 27; duration of illness could not be estimated for those with unknown or CHR diagnosis*.

Two of the 31 participants dropped out of the study prior to attending any MILO sessions. The remaining 29 attended at least three sessions of MILO, and 27 fully completed the intervention.

All 31 participants completed baseline (pre-training) survey assessments and recorded skills demonstrations. The two participants who dropped out prior to participating in MILO sessions were not contacted for post-intervention assessments. Of the 29 who attended at least three sessions of MILO, 28 completed post-intervention surveys and recorded skill demonstrations.

### Satisfaction

Scores from the CSQ (sent to participants within one week of MILO completion) are displayed in Table 3. Twenty-five of the 28 participants (89%) who completed post-intervention assessments reported that they had used the skills they learned in the intervention when communicating with the IP. An incidental finding was that at least eight of the 29 caregivers who participated in MILO sessions recommended the study to a family member or other social contact, suggesting high client satisfaction.

**Table 3:** Participant satisfaction (*N* = 28).

**Item (response range for each is 0–3, with “0” representing poor satisfaction and “3” representing full satisfaction)**	**Mean (SD)**
How would you rate the quality of service you have received?	2.89 (0.31)
Did you get the kind of service you wanted?	2.57 (0.50)
To what extent has our program met your needs?	2.50 (0.58)
If a friend were in need of similar help, would you recommend our program to them?	2.93 (0.26)
How satisfied are you with the amount of help you have received?	2.71 (0.46)
Have the services you received helped you to deal more effectively with your problems?	2.68 (0.55)
In an overall, general sense, how satisfied are you with the service you have received?	2.86 (0.36)

The PI (EK) was the study clinician for 19 participants, while co-authors BD and AF were the study clinicians for four and six participants respectively. There were no between-clinician differences in MILO session attendance [*F*_(2)_ = 0.53, *p* = 0.596] or CSQ total scores [*F*_(2)_ = 1.03, *p* = 0.371].

Results from the thematic analysis of participants' written qualitative responses are displayed in Table 4. Themes that emerged were participants' enthusiasm for the MILO principles and skills, their desire for additional memory aids and practice opportunities so that they could feel more confident using MILO skills, and their eagerness and ability to implement their newly acquired skills with their teen/young adult children.

**Table 4:** Qualitative response themes (*N* = 28).

**Prompt**	**Identified theme (number of responses within this theme)**
What have you found most helpful about this program?	MILO skills (22) Motivational interviewing “spirit” (7) Expertise and/or empathy of facilitator (6) Individualized advice about a specific family situation (6) Role plays (6) Convenience of telehealth (1)
What changes would improve this program in the future?	Offer more sessions and practice opportunities (11) Change wording/response options in one or more questionnaire (4) No changes (3) Provide scripts or memory aids to help with skill implementation (3) Improve telehealth platform (2) Offer training in a group format (2) Expand to diagnoses beyond FEP (2) Provide more rationale for motivational interviewing (1)
What barriers to implementing the MILO skills did you experience?	No barriers (9) Limited contact with the individual with psychosis (7) Not enough training/practice (5) IP not responsive to attempts to use skills (4) Difficulty managing own emotions during conversations (3) Not enough time for longer conversations (1)

## Discussion

The results of this pilot feasibility study indicate that MILO is a feasible brief intervention for parents of individuals suffering from early-course psychosis. The pace of recruitment proceeded more quickly than anticipated. Clinical staff at FEP treatment centers supported the intervention by sharing information about the study with families, and as the study progressed, additional participants were referred to the study by past participants who encouraged others in their families, support groups, or social networks to participate.

All participants were parents of IP, and many expressed gratitude for a resource that they could access even though their children were refusing to participate in treatment. Parents of untreated or under-treated individuals with early course psychosis may represent an underserved constituency. Although a number of participants expressed optimism that the MILO skills could be useful in facilitating their loved one's connection with or adherence to psychiatric care, for many participants, this outcome was secondary to their general relief at receiving guidance that would reduce overall conflict and stress in their family relationships. Illustrating this sentiment, one participant wrote that “[It was helpful to] learn specific techniques for interacting in difficult conversations and situations to produce a different outcome, to empower my child to assume more control for decisions affecting her life, [and] to feel heard and understood by someone with a relevant skill set who encouraged me and gently challenged my thinking so I could shift and think about a situation in a different way.”

Retention over the course of the intervention was strong. No participants dropped out after one or two sessions. Ninety percent of consented participants completed at least three MILO sessions as well as pre- and post-intervention assessments. This is favorable relative to median drop-out rate for non-pharmacologic interventions in schizophrenia, which a 2017 meta-analysis estimated as 19% (36). Three participants requested and were granted a fifth session to obtain additional coaching on how to use MILO skills with the IP. This is notable in the context of the study design, in which participants were not reimbursed for attending study sessions.

Participant satisfaction with the MILO intervention was high. Twenty-six out of 28 participants who completed a post-intervention satisfaction survey reported that they would “definitely” recommend the service to a friend in need of similar help. Qualitative responses to satisfaction-related prompts described how participants appreciated acquiring concrete communication skills, learning the philosophy of MI, receiving individualized advice, practicing skills via role plays, working with MILO clinicians, and meeting via telehealth. Satisfaction may have been influenced not only by MILO session content but also by the fast turnaround from inquiry to enrollment to first MILO session. The MILO team prioritized responding to inquiries and making eligibility decisions quickly. This required some tolerance of uncertainty with regard to IP who did not have a well-established (e.g., via inpatient hospital record) diagnosis of a primary psychotic disorder.

The primary theme that emerged in participants' suggestions for strengthening the intervention was that they felt they needed more time to review and practice the skills. Some participants noted that the skills were difficult to remember, especially in stressful moments. In response, the author group is creating a short video series explaining and illustrating the skills, which caregivers can watch on demand to help them remember the skills, which will be publicly available when complete. Additionally, we may consider offering an optional fifth or even sixth session a few weeks or months after the four “core” sessions, so that caregivers can practice the skills again and discuss difficulties that may arise over time.

A weakness of this study is that non-Hispanic whites and individuals with college degrees are over-represented in the study sample. Overall, these groups tend to be over-represented in clinical trial samples in the U.S. (37–39); this trend was likely exacerbated by the use of telehealth as a modality. Individuals without college degrees may have encountered barriers such as lack of high-quality internet, devices compatible with video conferencing, or paid time off to seek out mental health support. In phase two of this study (pilot efficacy trial), study authors will partner with a FEP clinic that primarily serves under-represented minority groups and offer some MILO sessions in-person rather than via telehealth. Another inherent weakness of the study design is uncertainty regarding the accuracy of parent-reported diagnoses, treatment utilization, and adherence. Even co-parents who both participated in the study sometimes disagreed on their child's medication adherence. In some cases, this could be because co-parents staggered their participation by a month or more; in others, the divergence in their reporting may be due simply to their differing perceptions of the situation. In phase 2 of this study, the study team will ask participants representing multiple members of the same family to reconcile any divergent responses relevant to the IP's treatment history and utilization.

Recruitment and data collection for this study took place at the height of the COVID-19 crisis in the United States, from May through December 2020. This may have impacted the results in a few ways. First, the telehealth modality, which was not part of the original study design, was well-received by study participants and increased the pool of potential participants beyond the Boston metro area. Second, caregivers may have been especially interested in learning new strategies to address conflict during this time when many were sheltering in place with their families and experiencing unfamiliar stressors. Third, two participants disclosed that they contracted COVID during the course of their study participation but elected to remain in the study while they isolated and convalesced.

The next steps following this pilot feasibility study are to 1, implement small changes to the intervention recommended by phase one participants; 2, alter the recruitment strategy to obtain a more demographically diverse sample; and 3, move to a randomization design that will enable evaluation of the impact of MILO relative to a control condition. In phase two of this study, participants will be randomly assigned to either immediate MILO or a six-week waitlist condition, after which they will be offered MILO sessions, which will enable evaluation of intervention effects. Effects will be evaluated based on intent to treat analysis. Overall, MILO appears to be a highly feasible intervention that yielded strong retention and very high satisfaction among participating caregivers. Intentional consultation at multiple stages of intervention development and study design with a range of experts and individuals with lived experience likely contributed to the design of a feasible and well-received intervention and assessment battery.

## Data Availability Statement

The raw data supporting the conclusions of this article will be made available by the authors, without undue reservation.

## Ethics Statement

The studies involving human participants were reviewed and approved by Committee on Clinical Investigation Institutional Review Board Beth Israel Deaconess Medical Center. Written informed consent for participation was not required for this study in accordance with the national legislation and the institutional requirements.

## Author Contributions

EK was responsible for the study design, supervision of the study team, accurate reporting of data, and drafting this manuscript. HT, AS, MK, and KE contributed to intervention development, measure selection, and participant recruitment. BD and AF contributed to intervention design and interpretation of results. All authors have reviewed and approved this submission.

## Conflict of Interest

The authors declare that the research was conducted in the absence of any commercial or financial relationships that could be construed as a potential conflict of interest.
